# Using the SaTScan method to detect local malaria clusters for guiding malaria control programmes

**DOI:** 10.1186/1475-2875-8-68

**Published:** 2009-04-17

**Authors:** Marlize Coleman, Michael Coleman, Aaron M Mabuza, Gerdalize Kok, Maureen Coetzee, David N Durrheim

**Affiliations:** 1 School of Animal, Plant & Environmental Sciences, University of the Witwatersrand, Johannesburg, Gauteng, South Africa; 2 Liverpool School of Tropical Medicine, Pembroke Place, Liverpool, L3 5QA, UK; 3 Mpumalanga Department of Health, 66 Anderson Street, Nelspruit, 1200, South Africa; 4 Vector Control Reference Unit, National Institute for Communicable Diseases, National Health Laboratory Service, 1 Modderfontein Road, Sandringham, 2131 Johannesburg, South Africa; 5 SA Research Chair in Medical Entomology & Vector Control, School of Pathology, University of the Witwatersrand, Johannesburg, South Africa; 6 Hunter New England Population Health and Hunter Medical Research Institute, Locked Bag 10, Wallsend, 2287, Australia

## Abstract

**Background:**

Mpumalanga Province, South Africa is a low malaria transmission area that is subject to malaria epidemics. SaTScan methodology was used by the malaria control programme to detect local malaria clusters to assist disease control planning. The third season for case cluster identification overlapped with the first season of implementing an outbreak identification and response system in the area.

**Methods:**

SaTScan™ software using the Kulldorf method of retrospective space-time permutation and the Bernoulli purely spatial model was used to identify malaria clusters using definitively confirmed individual cases in seven towns over three malaria seasons. Following passive case reporting at health facilities during the 2002 to 2005 seasons, active case detection was carried out in the communities, this assisted with determining the probable source of infection. The distribution and statistical significance of the clusters were explored by means of Monte Carlo replication of data sets under the null hypothesis with replications greater than 999 to ensure adequate power for defining clusters.

**Results and discussion:**

SaTScan detected five space-clusters and two space-time clusters during the study period. There was strong concordance between recognized local clustering of cases and outbreak declaration in specific towns. Both Albertsnek and Thambokulu reported malaria outbreaks in the same season as space-time clusters. This synergy may allow mutual validation of the two systems in confirming outbreaks demanding additional resources and cluster identification at local level to better target resources.

**Conclusion:**

Exploring the clustering of cases assisted with the planning of public health activities, including mobilizing health workers and resources. Where appropriate additional indoor residual spraying, focal larviciding and health promotion activities, were all also carried out.

## Background

Malaria is the most important parasitic disease of humans. Over three billion people live in malarious areas and the disease causes over 500 million cases with one to three million deaths per year [1,2]. An estimated one hundred million people in Africa are at risk of malaria epidemics [3]. In common with most vector-borne infectious diseases, malaria is heterogeneous in its distribution in time and space [4-6], and incidence can vary greatly between districts, towns and villages. This heterogeneity is affected by patterns of malaria vector distribution, human-vector contact, human host behavioural factors, house construction, and malaria prevention methods used [5,7-10].

Characterization of malaria heterogeneity may allow prioritization of risk areas and allow focused control interventions. The ability to identify localized malaria clusters in remote highland areas of east Africa facilitated early intervention in the absence of early warning systems, as these malaria "hotspots" remained constant in epidemic and non epidemic years [11].

A number of models have been developed for describing malaria spatial distribution and seasonality [12-15] transmission [8,16-18], mosquito distribution [19,20] and risk factors associated with malaria [21-23]. However many malaria endemic environments are resource limited and tools to assist decision support need to take account of these limitations.

Malaria remains a public health problem in north-eastern South Africa, in the low altitude provinces of KwaZulu-Natal, Limpopo and Mpumalanga [24,25]. Malaria risk is low compared to other hyper- and holo-endemic areas of sub-Saharan Africa and naturally acquired immunity does not develop in the local population [26].

The burden of malaria is well described in this region as definitive diagnosis using rapid diagnostic tests (RDTs) and mandatory reporting of malaria cases is universally practised in the public health system, which manages the vast majority of malaria cases [27,28]. All confirmed malaria cases are entered into a computerized malaria surveillance system.

This paper reports on the analysis of malaria notification data from 2002–2005 from Mpumalanga Province to determine local clustering of cases and how that was used to direct local control efforts and enhance the district outbreak identification and response system [29].

## Methods

### Study area

Mpumalanga Province, which borders Mozambique and Swaziland, is a predominantly rural area with a population of approximately four million people. Malaria occurrence in this area is seasonal during the wet and humid months of October to April. Ninety-five percent of malaria cases are due to *Plasmodium falciparum *infection. This area has historically experienced malaria outbreaks and epidemics with relatively high mortality [29,30]. The main vector in this region is *Anopheles arabiensis *and vector control is predominantly by indoor residual spraying (IRS) with DDT [31,32].

**Figure 1:**
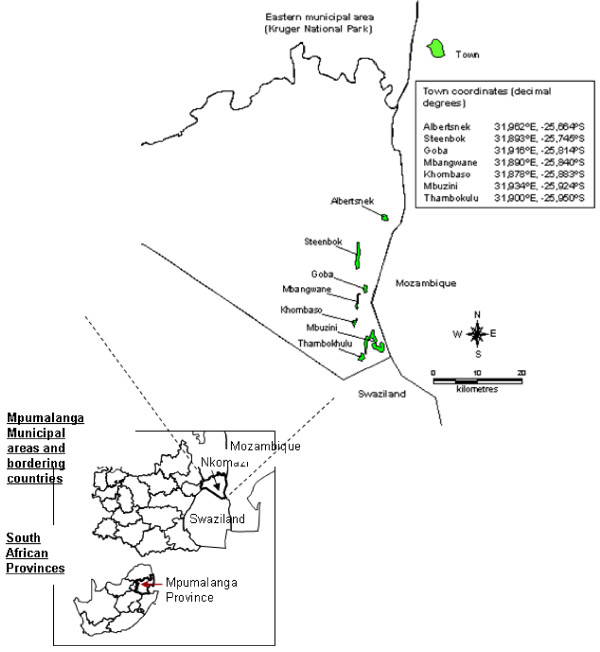
**Location of the seven towns selected for detailed cluster surveillance, Nkomazi municipal area, Mpumalanga**.

The seven rural towns that experience the highest malaria risk in Mpumalanga were included in this study (Figure 1)[33], with malaria incidence ranging from 30 to 61 per 1,000 persons between 1997 and 2005.

Original town maps acquired in 2000 were upgraded from digitized aerial photographs produced in 2002 (Cadnet, Nelspruit, South Africa) and updates were converted into MapInfo version 6.4 files (MapInfo Corporation, New York, USA) with Geomedia software (Symmetry Systems Inc., New York, USA). Unique stand (household) numbers were allocated during the digitizing process. A stand may include one or more structures belonging to a family unit on a designated piece of land.

### Active malaria case investigation

Malaria case investigators followed up all malaria cases reported at local health facilities for these towns as part of malaria control program activities for the 2002/2003, 2003/2004 and 2004/2005 seasons. A malaria case is defined as a person diagnosed either by positive rapid test or blood slide. An investigator would identify the probable source of infection by confirming the travel history of the patient recorded on the notification.

### Spatial and temporal clusters

SaTScan™ software, version 5.1.3, using the Kulldorf method of retrospective space-time permutation and the Bernoulli purely spatial model [34,35], was used by malaria control programme operational staff to detect malaria clusters in individual towns in the three seasons under investigation and over the combined time period. Individual malaria cases were used and recorded against source of infection households. Households who did not seek health care for malaria or tested negative during active case detection were used as controls for the Bernoulli method. More than one case can be reported per household. A household in these areas is typically defined as a family unit with a single land owner.

The circular scan statistic is isotopic with respect to the rotation of the geographical area . This method has previously been validated for plotting and understanding local malaria time-space-clusters [21,36-39].

Observed cases in a cluster were compared to the distribution of expected cases if spatial and temporal locations of all cases were independent. The model adjusts for entirely spatial or entirely temporal clusters. With spatial adjustment, time remained dormant and during temporal analysis seasons were considered. The distribution and statistical significance of the clusters were explored by means of Monte Carlo replication of data sets under the null hypothesis with replications greater than 999 to ensure adequate power for defining clusters . Clusters were prioritized for public health action according to their statistical significance. Hard copy town maps showing cluster areas were distributed to malaria field staff investigating cases allowing the coordination of intervention efforts within communities.

## Results

### Malaria incidence

**Figure 2:**
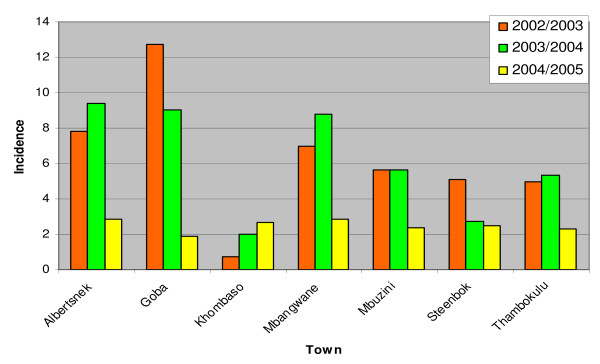
**Malaria case incidence per 1 000 population by town, 2002/2003 – 2004/2005 seasons, Nkomazi municipal area**.

Four hundred and twenty two malaria cases were notified during the three seasons from 341 households across the seven towns. Malaria incidence differed significantly between the towns during the three seasons (χ^2 ^= 6.442, p = 0.040) (Figure 2).

### Case clustering

**Figure 3:**
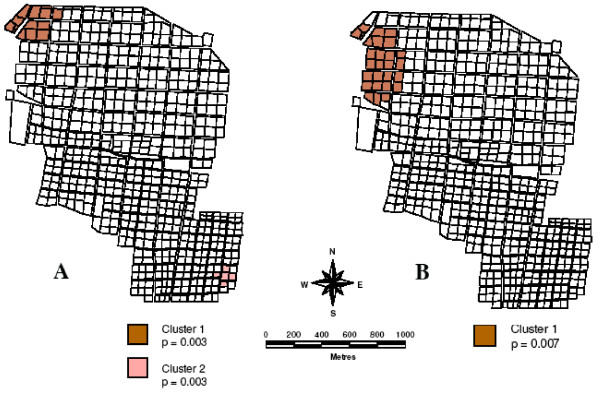
**Spatial malaria case clusters, Albertsnek town**. A. 2002/2003 – 2004/2005 seasons B. 2004/2005 season.

**Figure 4:**
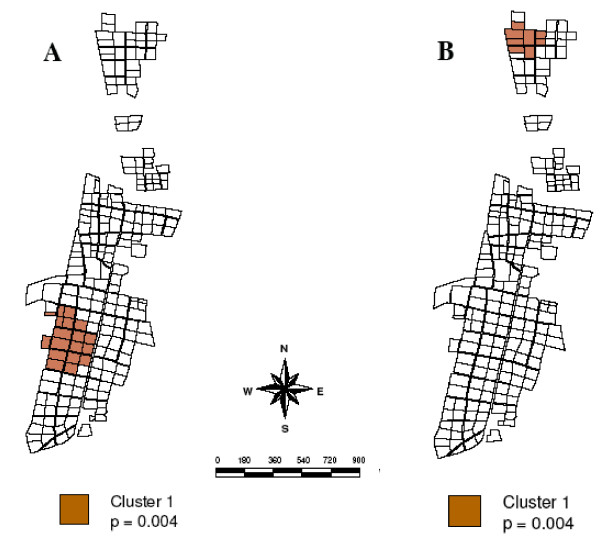
**Space time malaria case cluster, Thambokulu town**. A. 2004/2005 season. B. 2002/2003 – 2004/2005 seasons.

SaTScan analysis detected a number of clusters during the study period (Table 1). Albertsnek produced two space-clusters over the three-season period using the Bernoulli model, one in the northern (log likelihood ratio = 12.308, p = 0.003) and one in the south eastern part of town (log likelihood ratio = 12.187, p = 0.003) (Figure 3A). Three additional space-clusters were observed; in Goba (log likelihood ratio = 12.226, p = 0.001), Mbuzini (log likelihood ratio = 22.372, P = 0.001) and Thambokulu (Figure 4A) (log likelihood ratio = 22.372, p = 0.001). Only two towns, Albertsnek (test statistic = 5.548, p = 0.007) and Thambokulu (test statistic = 3.668, p = 0.004), had space-time clusters and both were during the 2004/2005 season (Figures 3B and 4B).

**Table 1:** Cluster and outbreak profile by town, 2002/2003 – 2004/2005 season

**Town**	**Number of space time** **clusters** **n (season)**	**Number of space** **clusters** **(2002/2003 to 2004/2005)**
Albertsnek	1 (2004/2005)	2

Goba	0	1

Khombaso	0	0

Mbangwane	0	0

Mbuzini	0	1

Steenbok	0	0

Thambokulu	1 (2004/2005)	1

### Cluster detection to guide control activities

The third season for case cluster identification, 2004/2005, overlapped with the first season of implementation of an outbreak identification and response system in the Nkomazi area [29]. Thambokulu and Albertsnek triggered outbreak declarations during this season (Table 1). Analysis demonstrated clusters in both towns during this season. Real time cluster detection, monitoring as cases occur, triggered at the same time as the binomial outbreak identification thresholds [29].

The physical plotting of cases by household during this season (2004/2005) as part of the cluster identification activities at district level and on hard copy maps provided a visual distribution of risk in the towns and assisted with the timely planning of public health activities especially logistics, including active case detection, early diagnosis and treatment of positive cases in the areas of the clusters, additional indoor residual spraying, focal larviciding and health promotion activities.

## Discussion

Application of the SaTScan method successfully identified malaria clusters and clearly demonstrated malaria risk heterogeneity at local level. Four towns in this study experienced spatial clusters and two produced space-time clusters in the 2004/2005 malaria season, the latter resulting in targeted local control efforts. The high rates of treatment-seeking behaviour at primary health care level in Mpumalanga [40] combined with the use of passive notification and active case detection, strengthens disease surveillance and provides good quality data for cluster identification. SaTScan cluster identification has also proven valuable for targeting control strategies in the Kenyan highlands [21]. Over a ten week period during a 2002 epidemic, spatial targeting of vector control interventions reduced the abundance of *Anopheles *mosquitoes. The investigation of causes for clusters within the areas reported here was not explored. The hypo-endemic nature of malaria in these areas does predict the unstable patterns of disease occurrence and more specifically outbreaks that can be clustered. The proximity of these areas to higher malaria risk areas such as Mozambique allows for the import of parasites and eventually local transmission.

Spatial clustering of infectious disease is enjoying renewed interest, particularly in areas of limited resources. Statistical methods have been used to investigate spatial clustering of dengue [41] encephalitis [19] and sleeping sickness but the application to malaria has been limited [42].

Gaudart *et al *[43] compared the oblique decision tree model, a complex statistical technique with Kulldorff's SaTScan™ cluster technique, which was used in this study. Gaudart *et al *[43] produced similar results using both methods in a village in West Africa to identify malaria risk clusters. This investigation confirmed the usefulness of the Kulldorff's scan statistic [44-46]. Due to the circular isotopic technique of Kulldorff's SaTScan™ it is a useful tool to detect clusters but has limitations on detecting irregular shaped clusters due to its fixed scan window [47,48].

The seven Mpumalanga towns under cluster surveillance were included in a malaria outbreak identification and response system based on formal case reporting [29]. There was strong concordance between recognized local clustering and outbreak identification in specific towns, with Albertsnek and Thambokulu both reporting malaria outbreaks in the same season as the time-space clusters. This synergy allows mutual validation of the two systems in confirming outbreaks, which demand additional resources, and cluster identification that could better target these resources within the affected town.

## Competing interests

The authors declare that they have no competing interests.

## Authors' contributions

MaCol carried out all the investigative work and analysis under the guidance of MCoe and DND Field work would not have been feasible without the assistance of AMM and GK MiCol drafted the manuscript and corrected subsequent drafts.
